# Tropolone and hinokitiol act against *Staphylococcus aureus* by concentration-dependent iron restriction or toxic iron overload

**DOI:** 10.1128/msphere.00619-26

**Published:** 2026-10-07

**Authors:** Bethany L. Hardman, Gary J. Sharples

**Affiliations:** 1 Department of Biosciences, Durham University 3057, Durham, United Kingdom; University of Galway, Galway, Ireland

**Keywords:** gram-positive bacteria, chelators, antibacterial agents, metal sequestration, iron, manganese, zinc, copper

## Abstract

Transition metal homeostasis is a critical component of bacterial physiology and thus represents a promising opportunity for antimicrobial intervention. Here, we demonstrate that the naturally occurring chelators tropolone (TRO) and hinokitiol (HNK) inhibit *Staphylococcus aureus* growth through concentration-dependent shifts in metal stress. Integrated metallomic and transcriptomic profiling revealed that low doses of both compounds promote toxic iron loading and a PerR-regulated oxidative stress response, consistent with iron-induced redox toxicity. However, at higher concentrations, both chelators trigger iron and manganese starvation responses, copper and zinc accumulation, and repression of respiratory and fermentative pathways, consistent with widespread disruption of metal homeostasis and failed metabolic adaptation. Despite these broadly similar responses, TRO, but not HNK, produced an unexpected biphasic growth phenotype. Experiments confirmed an analogous response in the distantly related gram-positive species *Bacillus subtilis*. These findings highlight how small molecule chelators can interfere with multiple metal systems to disrupt bacterial metabolism and how TRO can be exploited in a dose-dependent fashion to disrupt *S. aureus* proliferation.

**IMPORTANCE:**

There is an urgent need to develop alternative therapies to combat the drug-resistant pathogen *Staphylococcus aureus*. One solution is to disrupt the intricate balance of metal availability using small-molecule chelators. We show that tropolone and hinokitiol arrest bacterial growth at low and high doses by radically different mechanisms. At low concentrations, both chelators induce iron overload, whereas at higher concentrations, access to iron and manganese is blocked, and instead, toxic levels of intracellular copper accumulate. Despite the similarity in their cellular effects, only tropolone confers bacterial growth restriction at low and high doses, while permitting recovery at intermediate levels. Such contrasting concentration-dependent antibacterial activities could be harnessed, by iterative switching of doses, to radically perturb metal metabolism as a unique means to control *S. aureus* infections.

## INTRODUCTION

Transition metals are essential micronutrients for bacterial physiology, serving as cofactors in enzymes that drive multiple cellular processes (1, 2). While essential at physiological concentrations, excess metal accumulation can be deleterious. Ferrous iron can catalyze the Fenton reaction, generating reactive oxygen species (ROS) that damage nucleic acids, lipids, and proteins (3, 4). Excess copper and zinc can promote mismetallation of metalloproteins, disrupt Fe-S clusters, and promote oxidative stress (5, 6). Thus, bacterial survival depends on maintaining an optimal intracellular concentration for each metal (7, 8). Humans exploit this vulnerability through nutritional immunity, which involves manipulating metal availability during infection (2). Iron, manganese, and zinc are sequestered by host proteins to restrict microbial growth, and toxic metals like copper and zinc are exploited by actively mobilizing and concentrating them within phagolysosomes to kill engulfed pathogens (2). To counteract these pressures, bacteria have evolved sophisticated regulatory networks that coordinate metal acquisition, storage, utilization, and efflux (1). These systems rely on metal-sensing transcriptional regulators that precisely tune gene expression to intracellular metal availability (9).

*Staphylococcus aureus* is both a harmless commensal and serious pathogen, with a propensity to develop problematic drug resistance (10–12). To overcome iron restriction, *S. aureus* synthesizes the siderophores, staphyloferrin A and B, which chelate ferric iron and facilitate uptake through specific transport systems, HtsABC and SirABC (13, 14). This process is tightly controlled by the ferric iron uptake regulator, Fur (13). For manganese acquisition, *S. aureus* utilizes the MntABC and MntH manganese importers, both regulated by MntR (13, 15). Copper is not considered to be an essential micronutrient for *S. aureus*, and it employs a copper detoxification system (CopAZ), regulated by the copper-sensitive operon regulator, to export excess intracellular copper and avoid toxicity (13, 16). Notably, iron homeostasis intersects intimately with oxidative stress pathways, so the peroxide-responsive repressor, PerR, uses iron availability to monitor and modulate antioxidant defenses (17, 18). Iron is therefore both essential and toxic, and an intracellular balance is intricately linked to bacterial stress.

The centrality of bacterial metal homeostasis presents an attractive therapeutic vulnerability (19). Small molecule chelators that perturb intracellular metal availability, either by inducing metal starvation (20) or promoting toxic metal accumulation (19, 21, 22), offer potential as a fresh antimicrobial strategy (19). Tropolone (TRO) and derivatives, including hinokitiol (HNK), represent a structurally distinct class of metal chelators with broad biological activity (23). The tropolone motif consists of a seven-membered non-benzenoid aromatic ring bearing adjacent hydroxyl and carbonyl functional groups, which together form a bidentate metal-binding motif (24, 25). Although quantitative binding constants for tropolone-metal complexes are limited, available evidence suggests a general metal-binding preference of Fe^3+^ > Cu^2+^ > Zn^2+^ > Mn^2+^ > Mg^2+^ > Ca^2+^ (26). Tropolones exhibit bacteriostatic and bactericidal activities against a broad range of gram-negative and gram-positive bacteria (23, 24). Several lines of evidence point to a metal-dependent mechanism for the antimicrobial action of tropolones. Tropolone derivatives inhibit zinc-dependent enzymes by coordinating the catalytic zinc ion (27–29). In some bacterial species, tropolone-like molecules are produced as siderophores, suggesting potential roles in iron sequestration (30, 31). Indeed, an iron-restrictive mechanism has been demonstrated against *Escherichia coli (31*). Contrastingly, TRO and HNK can act as iron-ionophores, capable of shuttling the metal across biological membranes and promoting iron-induced oxidative stress (32, 33). HNK has attracted increasing interest as a therapeutic candidate due to its diverse biological activities and generally favorable preclinical safety profile (34). Consequently, a better understanding of the antibacterial mechanisms of TRO and HNK is required to inform future therapeutic development.

Our results reveal a concentration-dependent change in intracellular metal pools that cannot be explained solely by iron deprivation. At relatively low doses, both TRO and HNK promote toxic intracellular iron accumulation and induction of oxidative stress responses in *S. aureus*. However, at higher concentrations, both compounds cause a mechanistic shift toward functional manganese and iron starvation accompanied by copper accumulation and repression of core metabolic pathways. Interestingly, a biphasic growth inhibition phenotype was exhibited only with TRO, despite similarities in cellular response to both chelators; a similar inhibitory pattern has been noted previously but only with HNK (35). This work advances our understanding of metal-targeting small molecules as antimicrobial agents and establishes an integrated metallomic-transcriptomic framework that defines their mechanisms of action.

## RESULTS

### Differences in the antibacterial activity of TRO and HNK

**Fig 1:**
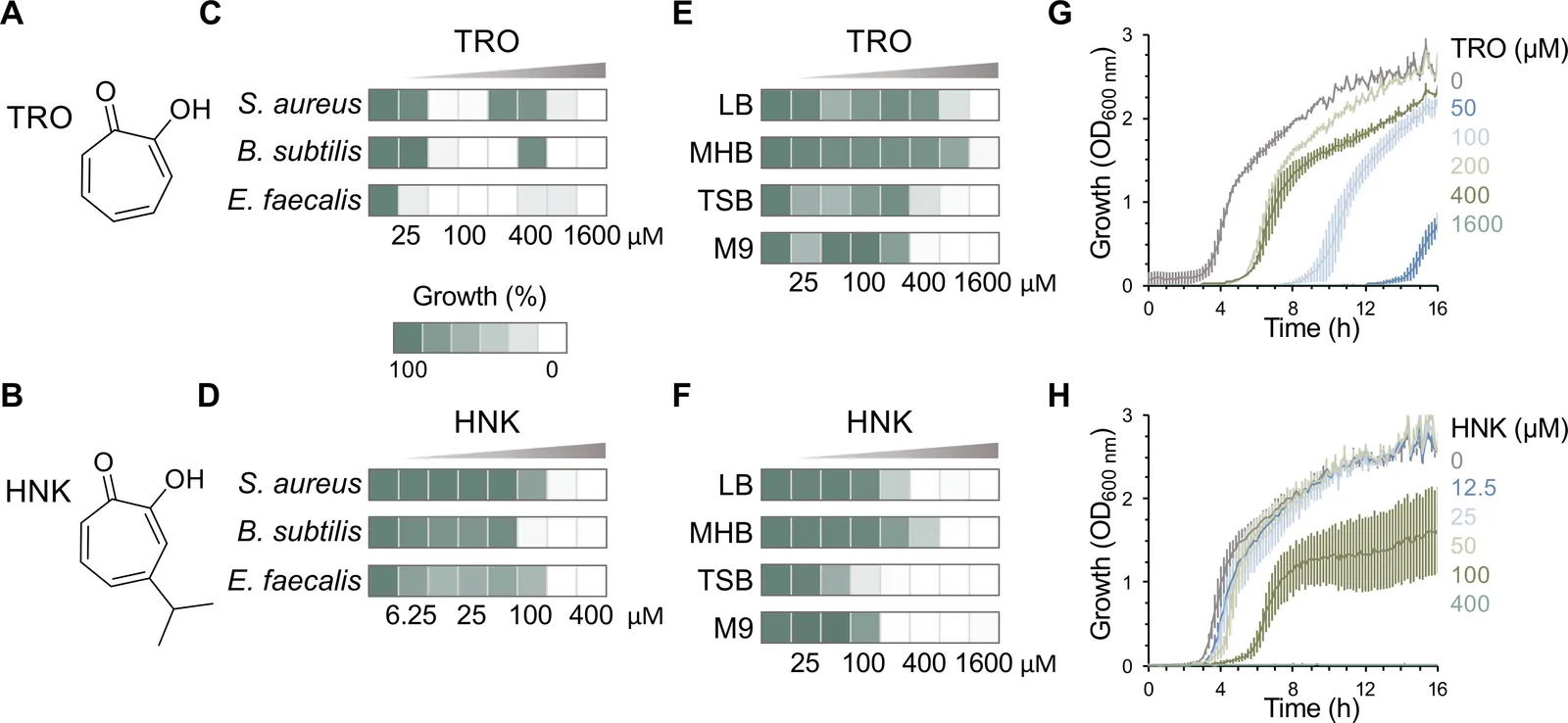
Inhibition of gram-positive bacterial growth by TRO and HNK. (**A and B**) Chemical structures of tropolone (TRO) and hinokitiol (HNK). (**C and D**) Susceptibility of *Staphylococcus aureus* FDA209P, *Bacillus subtilis*, and *Enterococcus faecalis* to TRO and HNK in Luria-Bertani broth (LB, Lennox). (**E and F**) Antimicrobial activity of TRO and HNK against *S. aureus* FDA209P in different growth media: LB, tryptone soya broth (TSB), cation-adjusted Muller-Hinton broth (MHB), and M9 minimal media. For testing different growth media, the chelant dilutions and 1:10 bacterial dilutions were prepared with the desired test media, using overnight cultures and inoculum prepared in LB broth. MICs were defined as the lowest concentration needed to inhibit bacterial growth by >90% relative to untreated controls. (**G and H**) Growth of *S. aureus* FDA209P in the presence of TRO or HNK. Data show the mean and standard deviation of three biological replicates.

To assess the antibacterial activity of TRO (Fig. 1A) and HNK (Fig. 1B), growth inhibition assays in Luria-Bertani (LB) broth were performed with three gram-positive species, *S. aureus*, *Bacillus subtilis*, and *Enterococcus faecalis*. The two chelators inhibited the growth of all three species (Fig. 1C and D). However, treatment with TRO, but not HNK, produced a concentration-dependent, biphasic inhibition profile in *S. aureus* and *B. subtilis*; growth was inhibited at 50–100 and ≥800 µM TRO, yet not between 200 and 400 μM (Fig. 1C). *E. faecalis* displayed a greater susceptibility to TRO, although a subtle biphasic effect was detectable (Fig. 1C). In contrast, HNK produced a more typical dose-response growth inhibition profile and minimum inhibitory concentrations (MICs) at 100–200 µM (Fig. 1D).

To establish whether the composition of the culture medium contributed to the unusual antibacterial activity pattern seen with TRO, the sensitivity of *S. aureus* to both chelators was compared in LB, tryptone soya broth (TSB), Muller-Hinton broth (MHB), and M9 media (Fig. 1E and F). *S. aureu*s showed similar susceptibility to TRO and HNK in the different media, although both compounds were slightly more active in TSB and M9 and less effective in MHB. The biphasic response with TRO was less obvious in MHB but pronounced in the other three media (Fig. 1E). Since the culture media did not substantially influence antibacterial efficacy, LB was selected for further experiments, allowing comparisons with previous studies (20, 36).

While the biphasic inhibition observed with TRO was fairly reproducible across independent experiments, the extent of growth inhibition within the lower concentration range showed some variability. Nevertheless, the characteristic pattern of growth inhibition at low and high concentrations of TRO, with recovery at intermediate concentrations, was consistently observed. Bacterial growth was therefore monitored over a 16 h period to gage how chelant exposure affects growth dynamics. Treatment with TRO at 50 μM extended the *S. aureus* lag phase to 14-15 h (Fig. 1G). This suggests that TRO is bacteriostatic against *S. aureus* at low concentrations and that cells require time to adapt before growth can resume. The length of the lag phase was gradually reduced at higher TRO concentrations (100–200 μM); the optical density of cultures treated with 200 μM TRO matched the untreated after 14 h growth (Fig. 1G). Bacterial growth was reduced with 400 μM TRO and completely eliminated at 1,600 μM (Fig. 1G). The results confirm that TRO differs in its antimicrobial efficacy against *S. aureus* at different concentrations, notably promoting growth at intermediate levels. In contrast, we observed a progressive reduction in bacterial growth with increasing concentrations of HNK (Fig. 1H).

### Effect of TRO and HNK on total cellular metal content

**Fig 2:**
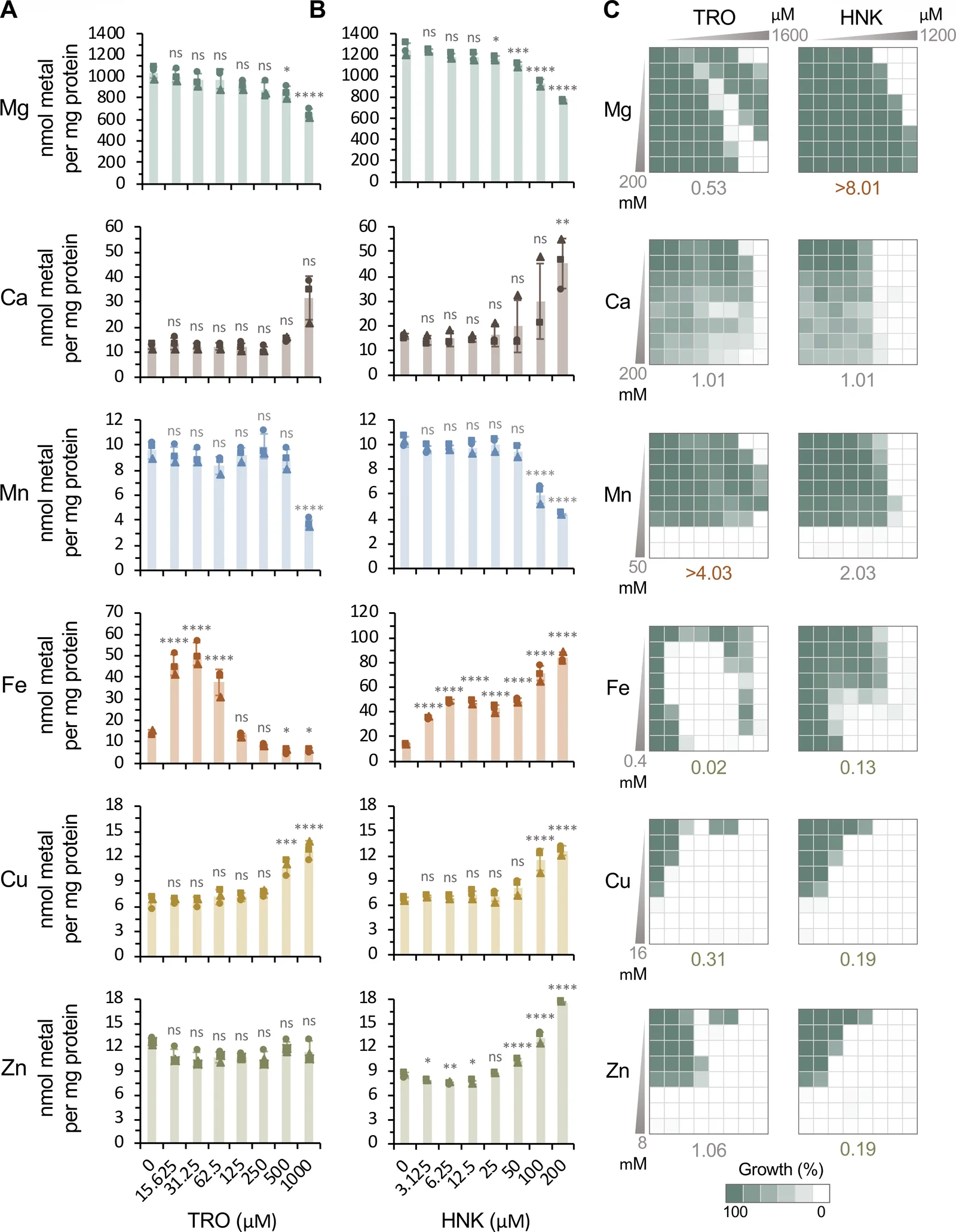
Influence of TRO and HNK on *S. aureus* metal homeostasis. (**A and B**) Effect of TRO and HNK on total cellular amounts of magnesium, calcium, manganese, iron, copper, and zinc in *S. aureus* FDA209P. Bacteria were cultured to early log phase in 20 ml of LB, in the presence or absence of chelant. Total cellular amounts of each metal were determined using ICP-MS and are presented as nmol metal per mg total cellular protein. A one-way analysis of variance (ANOVA) with a *post hoc* Dunnett test was performed to compare each chelant concentration against the untreated control (*n* = 3), *P < 0.05, **P < 0.01, ***P < 0.001, ****P < 0.0001, and ns = not significant. (**C**) Checkerboard assays were performed by growing *S. aureus* FDA209P with different combinations of TRO or HNK and metal salts. Data show the mean percentage growth, relative to untreated controls, of three biological replicates. Fractional inhibitory concentration (FIC) values were defined as the minimum concentration of compound needed to inhibit growth by ≥90% individually and in combination. Effects of metal-chelant combinations were identified as synergistic (≤0.5), indifferent (>0.5 to ≤ 4), or antagonistic (>4). The unusual pattern of some pairings and the inability to achieve an MIC for certain metal salts makes calculation of precise FIC indices challenging.

TRO and HNK are bidentate chelating agents, which bind metal ions through their cyclic ketone and enolic hydroxyl groups (24, 25). To understand how these two chelators influence *S. aureus* metal homeostasis, the cellular level of six biologically relevant metals was determined by inductively coupled plasma mass spectrometry (ICP-MS). Concentration-dependent changes in the cellular quantities of these metals were evident following exposure of *S. aureus* to either TRO or HNK (Fig. 2A and B). At lower concentrations, both chelators caused a significant increase in cellular iron (*P* < 0.0001). Treatment with higher concentrations of TRO caused a reduction in iron (Fig. 2A, *P* < 0.05), whereas exposure to higher concentrations of HNK caused a further increase in iron (Fig. 2B, *P* < 0.0001). Higher doses of both chelants also caused a reduction in manganese and an increase in copper, as well as changes in calcium and magnesium (Fig. 2A–B). Zinc levels were elevated by HNK at higher concentrations, a feature not apparent with TRO (Fig. 2A and B). Remarkably similar changes in cellular metal levels were noted with both TRO and HNK in *B. subtilis*, confirming the responses are not unique to *S. aureus* (Fig. S1A and B). Overall, the results are consistent with disruptions in bacterial metal homeostasis that vary with chelant concentration and potentially correspond to the observed biphasic growth restriction seen with TRO.

### Antibacterial efficacy of TRO and HNK in combination with metal salts

To gain further insight into the importance of metals in the antibacterial activity of TRO and HNK against *S. aureus*, checkerboard assays were performed with salts of each metal. The combination of TRO or HNK with FeSO_4_, CuSO_4_, or ZnSO_4_ yielded synergistic interactions, although the pattern with FeSO_4_ is atypical (Fig. 2C). MnSO_4_ produced antagonistic effects with both chelators, particularly with TRO, indicating that manganese supplementation partially alleviates growth inhibition caused by the chelators (Fig. 2C). MgSO_4_ generated an antagonistic interaction with HNK and shifted the biphasic growth inhibition pattern and the final MIC to higher concentrations of TRO, also indicative of antagonism (Fig. 2C). Combinations of TRO or HNK with CaCl_2_ produced an indifferent effect (Fig. 2C).

### Changes in *S. aureus* gene expression following TRO and HNK treatment

**Fig 3:**
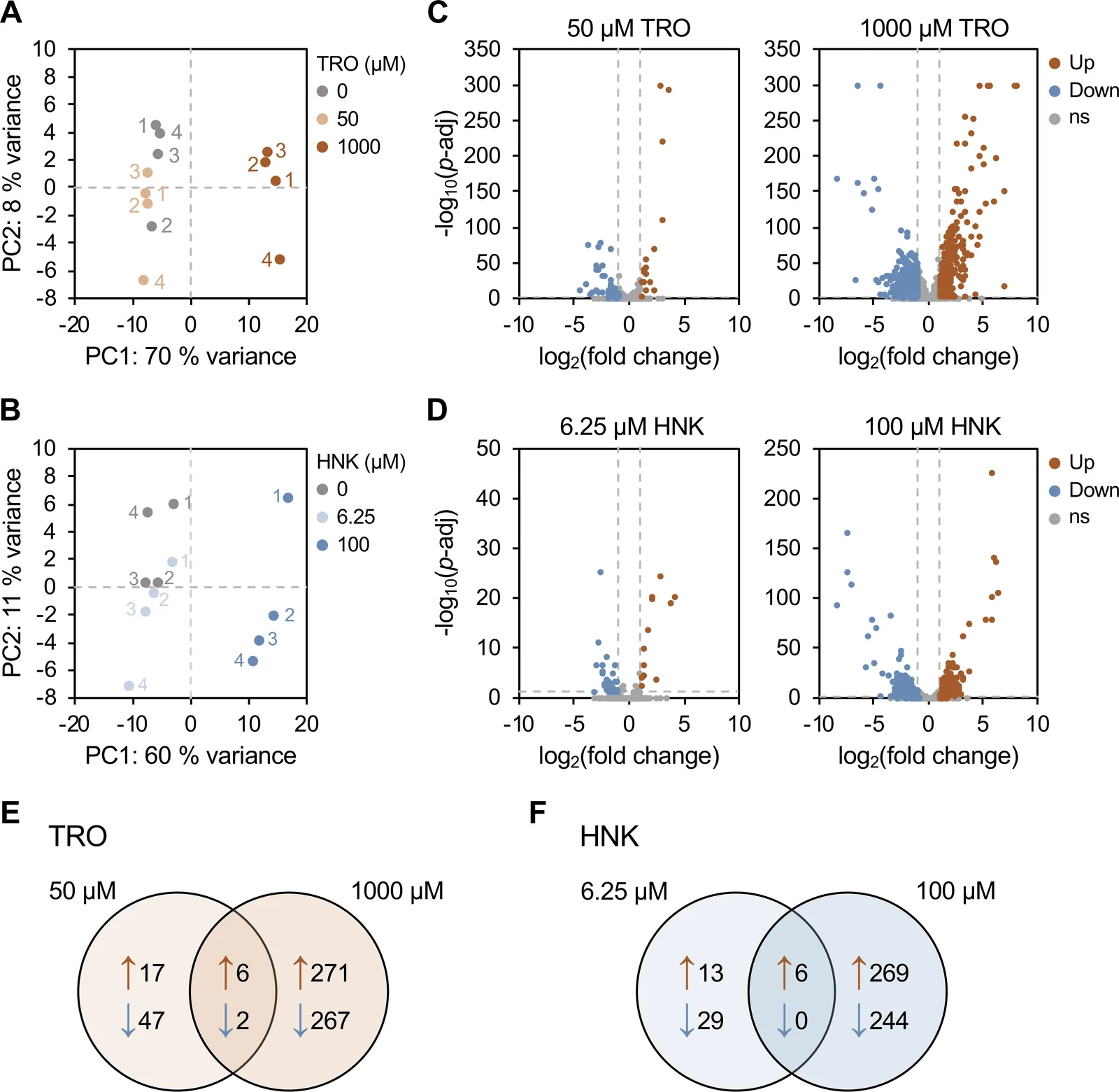
Transcriptional response of *S. aureus* treated with low and high concentrations of TRO and HNK. (**A and B**) Principal component analysis of DESeq2 normalized read counts for samples (*n* = 4) exposed to TRO and HNK. (**C and D**) Volcano plots of differentially expressed genes (DEGs) based on RNA-seq analysis of untreated and chelator-treated *S. aureus* FDA209P in early log phase for 30 min. Bacteria were cultured in 5 mL LB and treated with 50 and 1,000 µm TRO or 6.25 and 100 µM HNK. Upregulated genes are shown in orange (fold change > 2, *P*-adj < 0.05), downregulated genes are shown in blue (fold change < 0.5, *p*-adj < 0.05), and genes with no statistically significant difference in expression (ns = not significant) are shown in gray. (**E and F**) Venn diagrams showing the overlap between genes differentially expressed at the lower and higher concentrations of TRO and HNK.

The effect of chelant exposure on the transcriptome of *S. aureus* was investigated by RNA sequencing (RNA-seq). Low and high doses of TRO and HNK were examined to assess concentration-dependent transcriptional responses. Principal component analysis (PCA) of normalized RNA-seq data showed clear separation of samples by chelant concentration along PC1 (60%–70% variance), indicating that chelant treatment causes substantial changes in gene expression (Fig. 3A and B). PCA revealed partial overlap between untreated samples and samples exposed to 50 μM TRO or 6 μM HNK, suggesting that transcriptional differences between these two groups are subtle at the global level (Fig. 3A and B). Biological replicates clustered closely together, indicating good reproducibility within groups.

Differentially expressed genes (DEGs) were defined as those with adjusted *P*-values ≤0.05 and |log₂(fold change)| >1. Exposure to 50 μM TRO resulted in only 64 DEGs, including 17 upregulated and 47 downregulated genes (Fig. 3C). Treatment with 1,000 μM TRO, in contrast, produced 538 DEGs (271 upregulated and 267 downregulated; Fig. 3C). Only six upregulated and two downregulated genes were shared between the two TRO treatments, indicating that low and high concentrations of TRO trigger distinctly different transcriptional responses (Fig. 3E).

HNK produced a similar dose-dependent pattern (Fig. 3D). Treatment with 6 µM HNK yielded 42 DEGs (13 upregulated and 29 downregulated), whereas 100 µM HNK resulted in 513 DEGs (269 upregulated and 244 downregulated). As with TRO, overlap in DEGs between concentrations was minimal, with only six shared upregulated genes and none downregulated in common (Fig. 3F). Together, these findings are consistent with the chelators being responsible for highly concentration-specific transcriptional responses.

At low concentrations, both TRO and HNK elicited highly overlapping transcriptional profiles. Most upregulated genes belonged to the PerR regulon, including those for peroxide defense enzymes (*ahpCF* and *katA*), iron-storage proteins (*ftnA* and *dps*), and the ferric uptake regulator (*fur*) (Table 1) (37). This is consistent with Gene Ontology (GO) enrichment analysis, which identified hydrogen peroxide catabolic processes, response to reactive oxygen species, and cellular oxidant detoxification as significantly enriched biological processes for upregulated DEGs (Table S3). In contrast, downregulated genes included many members of the Fur regulon (Table 2), in keeping with an ~2.6-fold increase in *fur* expression. The repressed genes included multiple iron uptake systems, such as the ferric hydroxamate transporter (*fhuCB*), catechol-type siderophore transporter (*sstABCD*), and ferrous iron transport system (*feoAB*) (Table 2) (14). Also, significantly enriched GO biological processes for downregulated DEGs included siderophore-iron import and haem transport (Table S4). We also observed a modest induction of SOS and DNA repair genes with low TRO, but not HNK, including *recA*, *uvrAB*, *lexA*, and *sosA*, indicative of mild DNA stress (Table 1). Collectively, this pattern suggests that low chelant concentrations produce iron overload, followed by induction of oxidative stress responses, likely a consequence of activated Fenton chemistry (4, 38).

**TABLE 1:** The most upregulated DEGs in *S. aureus* FDA209P following treatment with 50 μM TRO or 6.25 μM HNK

ID^*a*^	Gene	Annotation	lLog_2_(fold change)	
			TRO	HNK
Oxidative stress response				
01755	*ahpF*	Alkyl hydroperoxide reductase subunit F	2.95	2.16
01760	*ahpC*	Alkyl hydroperoxide reductase subunit C	2.87	2.03
06545	*katA*	Catalase	2.24	1.61
Iron storage				
09690	*ftnA*	Ferritin	3.63	2.76
10965	*dps*	DNA starvation/stationary phase protection protein	3.08	1.32
Iron-sulfur cluster assembly				
04225	*sufC*	Fe-S cluster assembly ATP-binding protein	1.44	1.17
04230	*sufD*	Fe-S cluster assembly ATP-binding protein	1.46	1.17
04235	*sufS*	Cysteine desulfurizer	1.45	1.24
04240	*sufU*	Fe-S cluster assembly protein	1.46	1.18
04245	*sufB*	Fe-S cluster assembly protein	1.34	0.86
Iron homeostasis				
07430	*fur*	Ferric uptake regulator protein	1.39	1.24
Zinc homeostasis				
10995	*czrA*	Zinc-responsive transcriptional repressor	2.19	4.22
11000	*czrB*	Zinc efflux transporter	1.94	3.73
DNA damage response				
06580	*sosA*	SOS-induced cell division inhibitor	1.53	
09700		Putative exonuclease	1.28	0.89

^*a*^ SAFDA_RS.

**TABLE 2:** The most downregulated DEGs in *S. aureus* FDA209P following exposure to 50 μM TRO or 6.25 μM HNK

ID^*a*^	Gene	Annotation	Log_2_(fold change)	
			TRO	HNK
Iron homeostasis				
00350	*sirA*	Staphyloferrin B transporter substrate-binding protein	−3.01	−2.50
03180	*fhuC*	Ferrichrome transporter ATP-binding protein	−3.04	−2.04
03185	*fhuB*	Ferrichrome transport permease protein	−2.78	−1.91
03655	*sstA*	Catechol transporter permease protein	−3.90	−2.33
03660	*sstB*	Catechol transporter permease protein	−2.92	−2.52
03665	*sstC*	Catechol transporter ATP-binding protein	−2.83	−2.49
03670	*sstD*	Catechol transporter substrate-binding protein	−2.88	−2.65
05345	*isdA*	Iron-regulated surface determinant protein A	−2.62	−1.33
05350	*isdC*	Iron-regulated surface determinant protein C	−3.35	−2.02
05355	*isdD*	Iron-regulated surface determinant protein D	−2.46	−2.33
05360	*isdE*	Iron-regulated surface determinant protein E	−1.94	−1.96
11210	*htsA*	Staphyloferrin A transporter substrate-binding protein	−3.83	−2.75
11750	*fhuD2*	Ferrichrome transporter substrate-binding protein	−2.37	−1.56
13150	*feoB*	Ferrous iron transporter protein B	−2.52	−2.13
13155	*feoA*	Ferrous iron transporter protein A	−4.60	−3.01

^*a*^ SAFDA_RS.

A dramatically different transcriptional response was observed with high concentrations of either TRO or HNK involving the induction of selected metal acquisition pathways (Table 3). Fur-regulated iron uptake systems were the most highly upregulated, notably the biosynthesis and export pathways for staphyloferrin A and B (*sfaABCD* and *sbnABCDEFGHI*) and their corresponding uptake systems (*htsABC* and *sirABC*) (Table 3) (14). Haem transport, siderophore biosynthetic processes, and siderophore-iron import into cells were significantly enriched biological processes for upregulated DEGs (Table S5). In addition to iron-related genes, high concentrations of the chelators also induced expression of pathways involved in manganese import (*mntABC*) and copper (*copAZ*) and zinc export (*czrAB*), indicating significant perturbations of intracellular metal homeostasis (Table 3).

**TABLE 3:** The most upregulated DEGs in *S. aureus* FDA209P after treatment with 1,000 μM TRO or 100 μM HNK

ID^*a*^	Gene	Annotation	Log_2_(fold change)	
			TRO	HNK
Manganese homeostasis				
03100	*mntC*	Manganese transporter substrate-binding protein	7.99	6.14
03105	*mntB*	Manganese transporter permease protein	7.83	6.25
03110	*mntA*	Manganese transporter ATP-binding protein	7.99	5.99
Iron homeostasis				
00350	*sirA*	Staphyloferrin B transporter substrate-binding protein	4.96	1.94
00355	*sbnA*	Staphyloferrin B biosynthesis protein	6.80	2.83
00360	*sbnB*	Staphyloferrin B biosynthesis protein	6.83	2.85
00365	*sbnC*	Staphyloferrin B biosynthesis protein	6.15	2.63
00370	*sbnD*	Staphyloferrin B biosynthesis protein	5.91	2.50
00375	*sbnE*	Staphyloferrin B biosynthesis protein	5.12	2.33
00380	*sbnF*	Staphyloferrin B biosynthesis protein	4.67	1.69
08630	*isdH*	Iron-regulated surface determinant protein H	4.94	2.18
Zinc homeostasis				
10995	*czrA*	Zinc-responsive transcriptional repressor	5.37	5.68
11000	*czrB*	Zinc efflux transporter	5.27	5.77
Copper homeostasis				
13190	*copA*	Copper efflux transporter	5.54	5.74
13195	*copZ*	Copper chaperone	4.65	5.09

^*a*^ SAFDA_RS.

At high doses, both TRO and HNK resulted in downregulation of *ftnA*, consistent with repression under iron-limiting conditions (Table 4) (17). Treatment with both chelators also suppressed central metabolism, including downregulation of the respiratory nitrate and nitrite reductases (*narGHJI* and *nirBD*) (39, 40), multiple fermentation pathways (*ldh1*, *pflAB*, *adhE*, and *ackA*) (41, 42), and the succinate dehydrogenase complex (*sdhABC*) (43), indicating reduced activity of both respiratory and fermentative energy-generating pathways (Table 4). This was evident from GO enrichment analysis for downregulated DEGs involving nitrate assimilation, anaerobic respiration, and various other metabolic processes (Table S6).

**TABLE 4:** The most downregulated DEGs in *S. aureus* FDA209P after incubation with 1,000 μM TRO or 100 μM HNK

ID^*a*^	Gene	Annotation	Log_2_(fold change)	
			TRO	HNK
Iron storage				
09690	*ftnA*	Ferritin	−4.59	−2.99
Nitrogen metabolism				
12295	*narK*	Nitrate/nitrite transporter	−6.67	−7.56
12320	*narJ*	Respiratory nitrate reductase delta subunit	−6.09	−7.24
12325	*narH*	Respiratory nitrate reductase beta subunit	−6.53	−7.53
12330	*narG*	Respiratory nitrate reductase alpha subunit	−8.43	−8.53
12335	*nasF*	Uroporphyrinogen-III C-methyltransferase	−4.61	−4.94
12340	*nirD*	Nitrite reductase small subunit	−5.04	−5.29
12345	*nirB*	Nitrite reductase large subunit	−5.35	−5.61
12350	*nirR*	Nitrite reductase transcriptional regulator	−6.76	−5.84
Galactose metabolism				
11295	*lacC*	Tagatose-6-phosphate kinase	−4.28	−2.36
15080	*lacB*	Galactose-6-phosphate isomerase	−4.71	−2.31
15085	*lacA*	Galactose-6-phosphate isomerase	−4.87	−2.16
Fermentation				
00915	*pflB*	Formate acetyltransferase	−4.45	−2.95
00920	*pflA*	Pyruvate formate lyase activating enzyme	−4.16	−3.16
01010	*ldh1*	L-lactate dehydrogenase	−3.86	−2.83

^*a*^ SAFDA_RS.

A small selection of DEGs involved in iron (*isdA*), manganese (*mntB*), and zinc (*adcA*) uptake, copper export (*copA*), and peroxide detoxification (*katA*) were examined by reverse transcriptase-quantitative polymerase chain reaction (RT-qPCR) to verify the transcriptomic data (Fig. S2). The fold change in gene expression closely matched that obtained by RNA-seq at low and high concentrations of TRO (Fig. S2A and B) and HNK (Fig. S2C and D), confirming the radical differences in dose response in these cellular response pathways.

### Susceptibility of *S. aureus* and *B. subtilis* mutants to TRO and HNK

**Fig 4:**
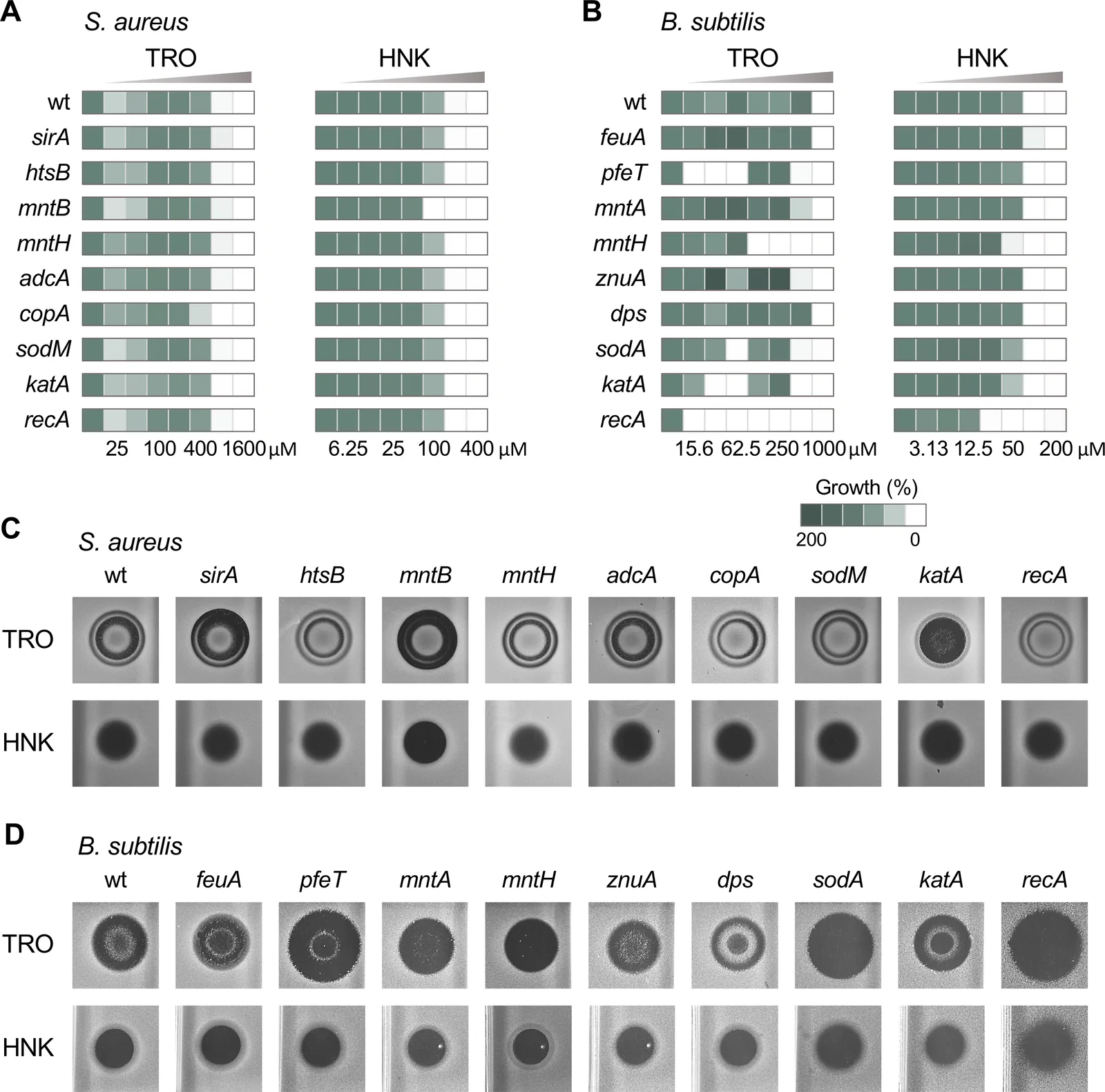
Susceptibility of *S. aureus* and *B. subtilis* mutants deficient in selected metal transport systems and the oxidative stress response to TRO and HNK. (**A and B**) *S. aureus* USA300 (*erm* insertions) and *B. subtilis* 168 (deletion-*kan* insertions) mutant strains were cultured in 100 µL of LB, in the presence or absence of TRO and HNK, by shaking at 37°C for 16 h. OD_600_ was recorded, and percentage growth was determined relative to untreated controls. Data show the mean percentage growth of three biological replicates (Data File S1). (**C and D**) Samples of TRO and HNK (20 mM, 10 µL) were applied on soft agar overlays containing *S. aureus* and *B. subtilis*, which were incubated at 30°C for 20 h. Lighter areas show bacterial growth and darker areas show growth inhibition. Representative images are shown, and two additional biological replicates produced similar results.

To investigate how disruptions in metal transport and stress response pathways influence susceptibility to TRO and HNK, MIC and antibacterial diffusion susceptibility assays were performed using selected transposon insertion mutants of *S. aureus* and deletion mutants of *B. subtilis*. Significantly, the biphasic response in liquid culture with TRO against both species (Fig. 4A and B) was also evident in overlay assays, with distinct rings of growth inhibition and secondary zones of recovery visible (Fig. 4C and D). This pattern was unique to TRO, with HNK producing only a typical single zone of bacterial growth inhibition (Fig. 4C and D). The assays also revealed important species-specific differences in susceptibility, highlighted in the appearance of inhibitory zones with TRO (Fig. 4C and D).

In *S. aureus* and *B. subtilis*, most mutants carrying defects in iron acquisition (e.g., *S. aureus sirA::erm* and *htsB::erm* and *B. subtilis* Δ*feuA::kan*) were no more susceptible to TRO or HNK than the wild type (Fig. 4A and B). However, the *B. subtilis* Δ*dhbA::kan* mutant showed no growth at 31 µM TRO (Fig. S3B). The *B. subtilis* Δ*pfeT::kan* mutant, defective in iron efflux, was hypersensitive to both low and high concentrations of TRO, but not HNK (Fig. 4B and D). Species-specific differences in susceptibility were also evident in cells deficient in manganese transport. The *B. subtilis* Δ*mntH::kan* mutant exhibited a lower MIC than the wt when treated with TRO and HNK (Fig. 4B), whereas the *B. subtilis* Δ*mneS::kan* and Δ*mneP::kan* mutants showed notably improved growth at 31 µM TRO (Fig. S3B). In contrast, only the *mntB::erm* mutant in *S. aureus* showed altered susceptibility, displaying increased sensitivity to HNK but not TRO in the MIC assays (Fig. 4A), yet greater inhibition with TRO in overlay assays (Fig. 4C). No zinc-related mutants in either species showed increased susceptibility to TRO or HNK, although several *B. subtilis* zinc-associated mutants experienced improved growth relative to the wt (Fig. S3).

Finally, mutants defective in oxidative stress and DNA repair pathways diverged in their responses between the two bacterial species. In *S. aureus*, none of the oxidative stress mutants showed altered growth in MIC assays (Fig. 4A), although *katA::erm* displayed growth inhibition with TRO on solid media (Fig. 4C). The *S. aureus* MHKAM (*sodA::Tn917 sodM::tet*) double transposon insertion mutant was more susceptible to high TRO concentrations but showed improved growth at lower concentrations of TRO (100 µM), and only mild sensitivity to HNK (Fig. S4A and B). However, growth of the *B. subtilis* Δ*katA::ery* and Δ*sodA::ery* mutants was completely inhibited at 62 and 500 µM TRO (Fig. 4B and D) and only mildly inhibited by 50 μM HNK (Fig. 4B). The *B. subtilis* Δ*recA::kan* mutant proved hypersensitive to TRO at all concentrations and was also inhibited by HNK (Fig. 4B and D). In contrast, the *S. aureus recA::erm* mutant experienced no reduction in growth (Fig. 4A and C).

The results with the different mutants reveal that susceptibility to TRO and HNK is strongly influenced by species-specific metal homeostasis and stress response pathways, with *B. subtilis* exhibiting broader sensitivity associated with disruptions in iron efflux, oxidative stress, and DNA repair pathways. In contrast, the selected *S. aureus* mutants displayed comparatively little susceptibility to either chelator.

## DISCUSSION

This study explored the antibacterial mechanisms of tropolone (TRO) and its isopropyl derivative, hinokitiol (HNK), against *S. aureus* using ICP-MS and RNA-seq to characterize changes in intracellular metal homeostasis and gene expression. The susceptibility of selected *S. aureus* and *B. subtilis* mutants defective in metal uptake and ROS detoxification helped validate these observations. While previous studies had suggested that TRO and HNK exert antibacterial effects by iron chelation (31), our findings reveal a previously uncharacterized dose-dependent effect on metal homeostasis. We demonstrate that low concentrations of TRO or HNK promote iron loading and oxidative stress, while higher concentrations induce iron and manganese starvation, coupled with copper and zinc accumulation. HNK elicited a broadly similar transcriptional response to TRO but lacked growth-inhibitory activity at lower doses, indicating important mechanistic differences despite shared metal-chelating properties.

**Fig 5:**
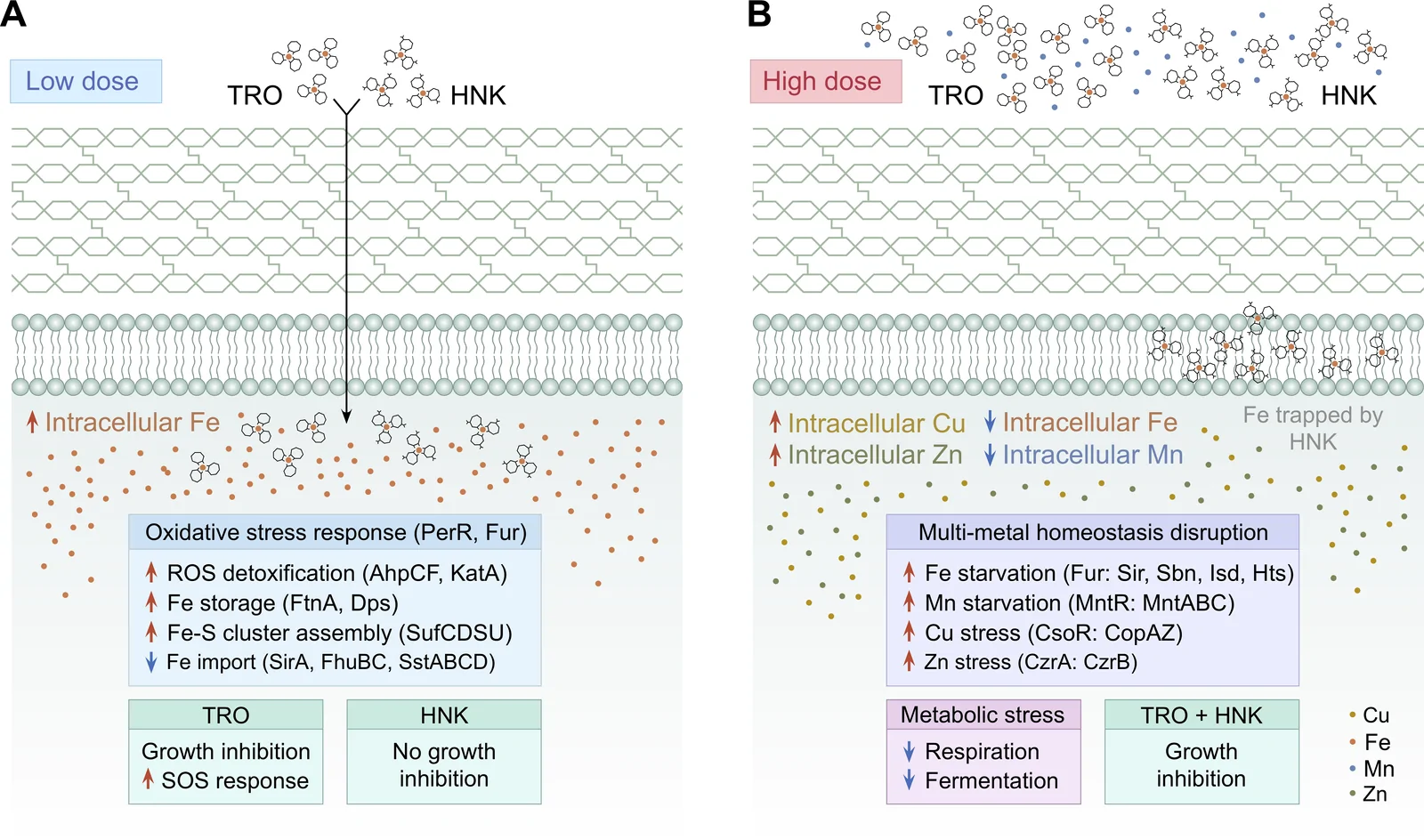
Concentration-dependent switch between opposing metal stresses in *S. aureus* exposed to TRO and HNK. (**A**) At low concentrations, TRO and HNK mediate Fe influx into the cell with accompanying cellular responses downregulating Fe import and upregulating iron storage proteins. Elevated intracellular Fe promotes oxidative damage, and thus, ROS detoxification pathways are activated, primarily via the PerR regulon. There are subtle differences between the action of TRO and HNK, with the former causing growth inhibition and induction of DNA damage response pathways that are not seen with HNK. (**B**) At high concentrations, TRO and HNK starve cells of intracellular Fe and Mn, while increasing Cu and Zn. This is apparent in the upregulation of Fe and Mn uptake systems and Cu and Zn export pathways. It is not clear how Mn is restricted or how Cu and Zn influx is mediated, although substantial Fe sequestration may allow the chelators to bind these other metals. Cell-associated iron concentrations increase with HNK, but not TRO, yet transcriptional responses suggest that both cause intracellular Fe restriction. The high Fe content could be due to HNK preferentially associating with the cell membrane as indicated. The disruption of normal metal homeostasis results in metabolic stress and growth inhibition by both TRO and HNK.

At low concentrations, both chelants produced a transcriptional signature consistent with increased intracellular iron (Fig. 5A). Upregulation of PerR-regulated oxidative stress genes and concurrent repression of Fur-regulated iron acquisition systems indicate an increase in the cytosolic labile iron pool, iron-dependent redox stress, and reduced demand for iron uptake. PerR functions as a peroxide-sensitive transcriptional repressor whose activity is modulated by intracellular iron and manganese levels (17). Similarly, Fur-mediated repression is relieved under iron limitation and enhanced under iron-replete conditions (14). Further evidence for oxidative stress was provided by induction of the *suf* operon, which encodes the sole Fe-S cluster assembly system in *S. aureus* (44). Fe-S clusters are highly sensitive to oxidative damage, and their oxidation releases additional iron into the cytoplasm, potentially amplifying redox imbalance (45). The hypersensitivity of *S. aureus* and *B. subtilis katA* (catalase) mutants fits with a reduced capacity to deal with peroxides in the presence of intracellular iron surplus. The observed increase in cellular iron levels supports this regulatory interpretation and suggests that in low doses, these chelators facilitate iron influx rather than depletion (Fig. 5A).

This interpretation is consistent with previous reports demonstrating that TRO and HNK can form membrane-permeable iron complexes that can shuttle iron across lipid bilayers (32, 33, 46). These lipophilic iron complexes enhance intracellular iron accumulation and ROS production in *S. aureus*, leading to complete growth inhibition under certain conditions (32, 33). Notably, this ionophore activity is highly dependent on the stoichiometry of the metal complex. In eukaryotic systems, HNK to iron ratios exceeding 3:1 do not generate ROS-induced DNA damage due to formation of a redox-inactive complex (47). Such stoichiometric effects may explain the apparent discrepancy between the variable responses highlighted here, complete growth inhibition observed previously (32, 33), and an aberrant pattern associated with HNK (35). Consistent with this interpretation, supplementation with iron enhanced chelant activity, suggesting that the degree of cellular damage depends on the balance of iron and chelator. Together, our findings extend existing models by demonstrating that low-dose TRO or HNK exposure in *S. aureus* promotes iron loading sufficient to activate oxidative stress responses and that this activity can be enhanced by optimizing the chelant-to-metal stoichiometry.

High concentrations of either TRO or HNK, in contrast, strongly induced expression of Fur-regulated iron acquisition pathways, including staphyloferrin biosynthesis and transport, which are canonical markers of iron limitation in *S. aureus* (14). This transcriptional response reflects a dose-dependent transition from iron influx at low concentrations to functional iron starvation at higher doses (Fig. 5A and B). Previous studies revealed that Fe(HNK)_3_ can aggregate at high concentrations, limiting the ability of HNK to mobilize iron across membranes (48). Such aggregation may reduce the efficiency of iron shuttling across lipid bilayers and contribute to iron starvation, although this mechanism has not yet been directly examined in bacteria. Notably, despite transcriptional responses consistent with iron limitation, total cellular iron levels increased following HNK exposure, but not with TRO. Because ICP-MS quantifies total metal content without distinguishing bioavailable from sequestered pools, these data suggest that a substantial fraction of HNK-associated iron is not physiologically accessible. Such differences in iron distribution may fit with structural differences between the two chelators; the isopropyl substituent unique to HNK could promote membrane association, trapping iron at the cell surface (Fig. 5B).

Beyond iron dysregulation, high-dose chelant exposure also affected transcriptional responses regulated by intracellular manganese, zinc, and copper levels (Fig. 5B). Upregulation of the manganese transporter *mntABC* indicated manganese starvation, while activation of *copAZ* and *czrAB* suggested intracellular copper and zinc excess. Manganese is critical for the activity of manganese-dependent enzymes, including superoxide dismutase and glycolytic enzymes such as phosphoglycerate mutase (GpmI) and fructose 1,6-bisphosphate aldolase (FbaA), and thus fulfills an important role in glycolysis and oxidative stress protection in *S. aureus* (15, 49, 50). Depletion of manganese may therefore compromise redox homeostasis and exacerbate metabolic stress induced by iron limitation. Indeed, supplementation with manganese significantly alleviated the toxicity of TRO against *S. aureus*. Interestingly, TRO and HNK exhibited slightly greater antibacterial activity in TSB and M9 media, both of which contain glucose, than in LB or MHB. As *S. aureus* preferentially utilizes glucose as a carbon source (51), this observation may be linked to the increased demand for manganese-dependent enzymes, including FbaA and GpmI (52, 53). Consequently, the manganese limitation induced by TRO and HNK may exert a greater metabolic burden during glucose utilization. Given the importance of glucose metabolism during host colonization and infection, this effect may have physiological relevance beyond laboratory growth conditions. Activation of *copAZ* and *czrAB* expression signals the accumulation of intracellular copper and zinc, metals that are tightly regulated due to their potential to disrupt protein folding and displace essential cofactors (13, 16, 54, 55). Copper can catalyze redox reactions and damage Fe-S cluster-containing enzymes, and both copper and zinc can competitively inhibit manganese- and iron-dependent proteins when present in excess (5). Addition of copper and zinc salts increased the antimicrobial activity of both chelators, possibly due to increased availability or uptake of the two metals, exacerbating intracellular metal toxicity. Thus, elevated copper and zinc stress responses suggest that chelant treatment interferes with metal availability, although the precise mechanisms of copper and zinc entry and toxicity require further investigation.

Importantly, high-dose chelant exposure was also associated with downregulation of respiratory genes, including components of the succinate dehydrogenase complex, and suppression of fermentative pathways. Iron-sulfur cluster-containing enzymes and manganese-dependent enzymes are central to respiratory metabolism, and their activities are highly sensitive to fluctuations in intracellular metal availability (56–58). Consistent with iron limitation, genes involved in nitrate respiration and the tricarboxylic acid cycle were downregulated, reflecting activation of the canonical iron-sparing response (59, 60). However, iron starvation in *S. aureus* typically triggers metabolic switching rather than complete metabolic repression, with increased reliance on fermentative pathways to sustain ATP production when respiratory enzymes are compromised (61, 62). In contrast, we observed concurrent suppression of both respiratory and fermentative genes, indicating that cells were unable to compensate metabolically. This pattern suggests that chelator exposure not only activates an iron-sparing response but instead induces a broader metabolic collapse. The simultaneous depletion of manganese, together with dysregulation of copper and zinc homeostasis, likely exacerbates enzymatic dysfunction beyond what might be expected under iron limitation alone.

Despite broadly similar transcriptional profiles, notable differences were apparent between TRO and HNK at low concentrations. TRO, but not HNK, induced modest expression of DNA repair genes, including the magnesium-dependent recombinase gene *recA*, and was associated with measurable growth inhibition. The selective induction of SOS-related genes by TRO suggests that it generates greater redox stress or more labile iron pools capable of promoting DNA damage, whereas HNK may impose oxidative stress that is below the threshold required to impair growth. Interestingly, the extent of growth inhibition observed at lower TRO concentrations varied somewhat between independent experiments. The prolonged lag phase observed in growth curve experiments suggests that this variability may reflect differences in the timing or efficiency of bacterial adaptation to chelator-induced stress, although the underlying mechanism remains unresolved. Structural differences between TRO and HNK may influence metal-binding affinity, redox potential, or cellular partitioning. These findings underscore that chemically related chelators can produce distinct physiological outcomes despite targeting overlapping metal pools. In addition, important species-specific differences were evident, with a *B. subtilis recA* mutant being substantially more susceptible to TRO than its counterpart from *S. aureus*. While ICP-MS measurements provide total cellular metal content, they do not distinguish between bioavailable, protein-bound, chelator-bound, or compartmentalized metal pools. Future studies employing metal-sensitive fluorescent probes or metalloproteomic approaches could help clarify metal distribution. Additionally, despite the limitations of such assays (63), direct measurement of ROS and DNA damage would further define the contribution of redox stress to growth inhibition.

The transition from iron loading at low concentrations to iron starvation at high concentrations represents a previously uncharacterized biphasic response. Bacterial growth at intermediate TRO concentrations likely reflects a delicate equilibrium between these opposing mechanisms of action. While chelator-mediated iron depletion has been widely proposed as the dominant antimicrobial mechanism of TRO and HNK in bacteria (23, 30, 31), we identify multi-metal homeostasis as an interconnected and exploitable vulnerability in *S. aureus*. Concurrent disruption of several metal regulatory networks reduces the likelihood of resistance emerging through single-pathway mutations. Previous studies have also demonstrated that TRO and HNK, or their iron complexes, can enhance the activity of several conventional antibiotics against methicillin-resistant *S. aureus* and other bacterial pathogens (32, 33, 64, 65). Given the widespread disruption of metal homeostasis observed in the present study, it will be important to determine whether these chelators can act as antibiotic resensitizing agents, either through potentiation of existing antimicrobials or through exploitation of metal-dependent vulnerabilities during infection. From a translational perspective, HNK has demonstrated a generally favorable safety profile in preclinical studies, whereas considerably less toxicology information is available for TRO. Although the concentrations examined here reveal distinct mechanisms of metal dysregulation, further work will be required to establish whether comparable antibacterial effects can be achieved within a clinically acceptable therapeutic window. Nevertheless, the potential to switch between low and high doses of these two chelators and cycle between metal overload and starvation may prove highly beneficial for therapeutic intervention.

## MATERIALS AND METHODS

### Bacterial strains, growth media, and compounds

Bacterial strains used in this study are listed in Table S1. *S. aureus* USA300 transposon insertion mutants were obtained from the Nebraska Transposon Mutant Library (66). *B. subtilis* mutants were constructed by transforming strain 168CA with genomic DNA from the *B. subtilis* deletion library (67), according to established methods (68). Culture media used were LB broth (Lennox; Sigma Aldrich), TSB (Millipore), MHB (Millipore), and M9 (1× M9 medium, 1 mM MgSO_4_ [VWR Chemicals], 0.2% glucose [Sigma Aldrich], and 0.1% casamino acids [Fisher Scientific]) (69). Na_2_HPO_4_ (30 g/L) (Sigma Aldrich), 15 g/L KH_2_PO_4_ (Fisher Scientific), 5 g/L NH_4_Cl (Sigma Aldrich), 2.5 g/L NaCl (Duchefa Biochemie), 15 mg/L CaCl_2_ (Sigma Aldrich), and 2.5 mg/L FeSO_4_ (Sigma Aldrich) were used to prepare a 5× M9 medium. All media were sterilized at 121°C for 15 min at 15 psi in a Dixons Vario 1528 autoclave.

TRO and HNK (β-thujaplicin) were dissolved in 100% DMSO and obtained from Merck, as were kanamycin, erythromycin, CuSO_4_, ZnSO_4_ and MnSO_4_.

### Minimum inhibitory concentration (MIC) assay and growth curves

MIC assays were performed using the broth microdilution method (70). Stock solutions of TRO or HNK, prepared in DMSO, were diluted in the desired culture medium and 50 µL transferred to a 96-well, round-bottomed plate (Sarstedt). Bacteria were grown at 37°C with shaking at 150 rpm to an optical density (OD_600_) of 0.07. Cultures were diluted 10-fold and 50 μL (1 × 10^6^ CFU/mL) used to inoculate the plate containing the chelant and DMSO controls. Plates were incubated for 16 h at 37°C with shaking at 150 rpm and the OD_600_ measured at the endpoint using a Spectrostar Nano plate reader (BMG Labtech). Time course experiments were performed to evaluate effects on *S. aureus* growth kinetics, with plates prepared as above and growth at 37°C monitored every 10 min.

### Antibacterial diffusion susceptibility assay

An LB soft agar overlay (0.6% agar, 7.5 mL) containing 200 µL of bacterial overnight culture was poured onto the surface of an LB agar plate. Chelators and DMSO controls (10 µL) were applied to the overlay and allowed to soak into the surface. Plates were incubated for 18–20 h at 30°C prior to visualization.

### Determination of cellular metal content

TRO or HNK were added to 20 mL LB broth in a 50 mL Falcon tube (Sarstedt) prior to inoculation with *S. aureus*. DMSO controls were set up in parallel. Cultures were grown at 37°C with shaking at 150 rpm for 2 h (OD_600_ 0.4–0.6). For similar experiments with *B. subtilis*, 20 mL cultures were grown to early-mid log phase (OD_600_ 0.3) before addition of chelator. Cells were pelleted by centrifugation and resuspended in 10 mL wash buffer (0.5 M sorbitol, 10 mM HEPES, pH 7.8). Cells were pelleted again and washed twice more. Cell pellets were digested in 1 mL 65% nitric acid (Supelco) for at least 16 h. Digests were diluted with 2.5% nitric acid and internal silver and bismuth standards (Sigma Aldrich) in a 1:8:1 ratio. A matrix-matched calibration curve was prepared using known quantities of metals in nitric acid (ICP multi-element standards, CertiPUR, Merck). Dilutions and a calibration curve were analyzed by ICP-MS (ThermoFisher iCAP RQ). Instrument control, analysis, and quantification used the PlasmaLab software interface (Thermo Scientific). Results are presented as nanomoles of metals per mg of cellular protein. Total cellular protein was measured using the Bradford assay on lysed *S. aureus* and *B. subtilis* cells.

### Checkerboard assay

Checkerboard assays were performed to determine the antibacterial efficacy of chelant-metal combinations (71, 72). Serial dilutions of chelator and metal salts were prepared in LB broth. Increasing concentrations of chelant were added to the 96-well plate horizontally, and increasing concentrations of metal salts were added vertically to create a checkerboard pattern. The inoculum was prepared as in MIC experiments. The plates were incubated for 16 h at 37°C with shaking (150 rpm), and the OD_600_ was measured.

### RNA extraction

Bacteria were cultured in 5 mL LB broth at 37°C with shaking to early log phase (OD_600_ 0.3). Cultures were treated with either TRO, HNK, or DMSO and incubated for a further 30 min (OD_600_ 0.4–0.5). Samples (1 mL) were mixed with 2 mL of RNAprotect Bacteria Reagent (Qiagen) to stabilize the RNA. Cell lysis was achieved by additions of lysozyme (15 mg/mL; Merck), lysostaphin (0.2 µg/mL; Merck), and proteinase K (>600 mAU/mL, Qiagen). RNA was extracted using an RNeasy Mini Kit (Qiagen), treated with DNase I (Fermentas), and RNA quality assessed with a Nanodrop (Thermo Fisher Scientific).

### Transcriptomics analysis

Library construction, RNA-seq, quality control, quantification, mapping, and statistical calculations were performed by Novogene. Briefly, ribosomal RNA was removed from total RNA and precipitated with ethanol. A library was constructed from the total RNA and sequenced using Illumina sequencing. Mapping to the reference genome was performed using Bowtie2 (73). Gene expression levels between treated and untreated samples (*n* = 4) were compared using the DESeq2 package in R studio (74) to determine the log_2_(fold change). The Benjamini-Hochberg procedure was used to control the false discovery rate and generate adjusted *P*-values (*P*-adj). Complete RNA-seq data sets are provided in Data File S1. Differentially expressed genes (DEGs) were mapped to Gene Ontology (GO) using enrichment analysis powered by PANTHER (75–77). A Fisher’s exact test with false discovery rate correction was used to determine which GO terms were significantly enriched (*P*-adj < 0.05) in each of the three GO components (76).

### Reverse transcriptase-quantitative polymerase chain reaction (RT-qPCR)

Transcriptional responses to changes in cellular metal levels and oxidative stress in *S. aureus* were validated by RT-qPCR. Following RNA extraction and DNase treatment, complementary DNA (cDNA) was generated using the ImProm-II Reverse Transcriptase System (Promega) with control reactions excluding reverse transcriptase. Transcript abundance was determined using the primers listed in Table S2. RT-qPCR analysis was conducted using 20 µL reactions containing 5 ng cDNA, 400 nM primer, and PowerUP SYBR Green Master Mix (Thermo Fisher Scientific). Each biological replicate (*n* = 3) was analyzed in technical triplicate using a Rotor-Gene Q 2plex (Qiagen, version 2.3.5). Cq values were determined with LinRegPCR (version 2021.1) after correcting for amplicon efficiency. Change in gene expression, relative to the control, was calculated using the 2^-∆∆Cq^ method and expressed as log_2_(fold change) (78).

### Statistical analysis and reproducibility

A one-way analysis of variance (ANOVA) with a *post hoc* Dunnett test was performed for multiple group comparisons with an untreated control (*n* = 3). The analyses were performed in Microsoft Excel or IBM SPSS Statistics (Version 29).

## Data Availability

The research data that supports our findings are included in the paper as figures, Supplemental material, and Data File S1.

## SUPPLEMENTAL MATERIAL

Data File S1 msphere.00619-26-s0001.xlsx Further details on the data summarized in the text, including replicates.

Supplemental Material msphere.00619-26-s0002.pdf Tables S1-S6; Figures S1-S4.

ASM does not own the copyrights to Supplemental Material that may be linked to, or accessed through, an article. The authors have granted ASM a non-exclusive, world-wide license to publish the Supplemental Material files. Please contact the corresponding author directly for reuse.
