# Supplementary material for: Tropolone and hinokitiol act against Staphylococcus aureus by concentration-dependent iron restriction or toxic iron overload
Source: mSphere. 2026 Oct 7;11(10):e00619-26. doi: 10.1128/msphere.00619-26 (PMC13711788; doi:10.1128/msphere.00619-26)
Supplement: Supplementary file 1 [file msphere.00619-26-s0002.pdf]

Supplemental material

Tropolone and hinokitiol act against *Staphylococcus aureus* by concentration-dependent iron restriction or toxic iron overload

Bethany L. Hardman and Gary J. Sharples

Department of Biosciences, Durham University, Durham, DH3 1LE, United Kingdom

TABLE S1 Bacterial strains used in this study.

Species	Strain	Source and genotype
Staphylococcus aureus	FDA209P, ATCC 6538P	Reference methicillin-sensitive S. *aureus* (MSSA) strain (1).
	JE2, USA300 LAC	Community-associated methicillin-resistant (CA-MRSA) parental strain for the NARSA collection. Obtained from the University of Nebraska via Kevin Waldron and Charles Winterhalter, Newcastle University.
	NE146	As JE2, but *sstA*::*erm*
	NE199	As JE2, but *czrB*::*erm*
	NE278	As JE2, but *zagA*::*erm*
	NE332	As JE2, but *isdA*::*erm*
	NE374	As JE2, but *feoB*::*erm*
	NE406	As JE2, but *fhuC*::*erm*
	NE548	As JE2, but *sfnA*::*erm*
	NE561	As JE2, but *copA*::*erm*
	NE568	As JE2, but *adcA*::*erm*
	NE805	As JE2, but *recA*::*erm*
	NE869	As JE2, but *htsB*::*erm*
	NE1097	As JE2, but *mntH*::*erm*
	NE1163	As JE2, but *iruO*::*erm*
	NE1172	As JE2, but *isdF*::*erm*
	NE1198	As JE2, but *sbnA*::*erm*
	NE1224	As JE2, but *sodM*::*erm*
	NE1363	As JE2, but *srtB*::*erm*
	NE1366	As JE2, but *katA*::*erm*
	NE1550	As JE2, but *ntrA*::*erm*
	NE1571	As JE2, but *ahpF*::*erm*
	NE1767	As JE2, but *sirA*::*erm*
	NE1784	As JE2, but *mntB*::*erm*
	NE1881	As JE2, but *fhuB*::*erm*
	SH1000	8325-4 RsbU$^+$ (2). From Henrik Strahl, University of Newcastle.
	MHKAM	As SH1000 but, *sodA*::Tn*917 sodM*::*tet* (3). From Henrik Strahl, University of Newcastle.
Bacillus subtilis	168CA	*trpC2* (4). From Henrik Strahl, University of Newcastle.
	BH343	As 168, but Δ*mneS::kan* Deletion library strain BKK06320
	BH334	As 168, but Δ*znuA::kan* Deletion library strain BKK02850

	BH328	As 168, but Δ*mntA::kan*<br>Deletion library strain BKK30770
	BH340	As 168, but Δ*mntH::kan*<br>Deletion library strain BKK04360
	BH331	As 168, but Δ*dps::kan*<br>Deletion library strain BKK30650
	BH352	As 168, but Δ*pfeT::kan*<br>Deletion library strain BKK13850
	BH355	As 168, but Δ*fhuD::kan*<br>Deletion library strain BKK33320
	BH358	As 168, but Δ*fecC::kan*<br>Deletion library strain BKK07520
	LJ165	As 168, but Δ*zagA::kan*<br>Deletion library strain BKK03360
	LJ168	As 168, but Δ*dhbA::kan*<br>Deletion library strain BKK32000
	LJ172	As 168, but Δ*cadA::kan*<br>Deletion library strain BKK33490
	BH325	As 168, but Δ*efeU::kan*<br>Deletion library strain BKK38280
	BH337	As 168, but Δ*yfiY::kan*<br>Deletion library strain BKK08440
	BH346	As 168, but Δ*yxeB::kan*<br>Deletion library strain BKK39610
	BH349	As 168, but Δ*mneP::kan*<br>Deletion library strain BKK05470
	LJ166	As 168, but Δ*feuA::kan*<br>Deletion library strain BKK01630
	LJ173	As 168, but Δ*sodA::ery*<br>Deletion library strain BKE25020
	LJ170	As 168, but Δ*sodF::kan*<br>Deletion library strain BKK19330
	LJ145	As 168, but Δ*katA::ery*<br>Deletion library strain BKE08820
	LJ171	As 168, but Δ*katE::kan*<br>Deletion library strain BKK39050
	LJ169	As 168, but Δ*ohrA::kan*<br>Deletion library strain BKK13140
	LJ167	As 168, but Δ*recA::kan*<br>Deletion library strain BKK16940
Enterococcus faecalis	NC 12697,<br>ATCC 29212	Reference strain, National Collection of Type Cultures,<br>Public Health England

TABLE S2 Oligonucleotides used for RT-qPCR with *S. aureus* in this study.

Primer name	Nucleotide sequence (5'-3')	Length	%G+C	Product (bp)
*isdA*_F	ACAGAAGCTACGAACGCAAC	20	50	108
*isdA*_R	GTGTGACTTCTCTGAAGAGC	20	50	
*mntB*_F	AGGGATAGTTTGTGGTACAG	20	45	108
*mntB*_R	AAGAATGATAGGGCAACACC	20	45	
*mntH*_F	CAACAATGGGTGCAATCATG	20	45	106
*mntH*_R	CCTTCCATTACAATTTGTCC	20	40	
*adcA*_F	CGCATGTGGGAATGATGATG	20	50	111
*adcA*_R	ACCTTCACGTGTTTTCCACC	20	50	
*copA*_F	TGAACACCCATTGGCAGAAG	20	50	109
*copA*_R	CGTTGCTTCAATACCATGGC	20	50	
*katA*_F	AATAGTATGACAGCAGGGCC	20	50	110
katA_R	GCATGCATTCGACGTTCTGG	20	55	
*rpoD*_F	ACAGGACTTAGGTCGAGATC	20	50	108
*rpoD*_R	AATGAAACAGGTTCTTGCGC	20	45	

Control reactions without a cDNA template were run for each primer pair alongside control reactions without reverse transcriptase for the reference gene primer pair (*rpoD*).

TABLE S3 Significantly enriched GO terms (*p*-adj <0.05) of upregulated DEGs after treatment of *S. aureus* with low chelant doses (50 µM TRO or 6.25 µM HNK).

Annotation	Fold enrichment	
	TRO	HNK
GO Biological Process		
Hydrogen peroxide catabolic process	>100	>100
Iron-sulphur cluster assembly	97.11	95.24
Response to reactive oxygen species	67.98	88.89
Cellular oxidant detoxification	33.99	44.45
Cellular homeostasis	20.60	26.94
GO Molecular Function		
Oxidoreductase activity, acting on metal ions	>100	>100
Ferric iron binding	>100	>100
Ferrous iron binding	>100	>100
NADH-dependent peroxiredoxin activity	>100	>100
GO Cellular Component		
Iron-sulphur cluster assembly complex	>100	>100

TABLE S4 Significantly enriched GO terms (*p*-adj <0.05) of downregulated DEGs after treatment of *S. aureus* with low chelant doses (50 µM TRO or 6.25 µM HNK).

Annotation	Fold enrichment	
	TRO	HNK
GO Biological Process		
Siderophore-iron import into cell	43.96	61.91
Haem transport	41.87	68.79
Transmembrane transport	3.39	3.83
Primary metabolic process	0.16	<0.01
Biosynthetic process	0.10	-
Macromolecule metabolic process	<0.01	-
Cellular response to iron ion	-	41.27
GO Molecular Function		
Transmembrane transporter activity	3.59	4.05
GO Cellular Component		
Outer membrane-bounded periplasmic space	20.93	34.39
Plasma membrane	2.88	3.15
Cytoplasm	-	<0.01

TABLE S5 Significantly enriched GO terms (*p*-adj <0.05) of upregulated DEGs in *S. aureus* following exposure to high concentrations of chelant (1000 μM TRO or 100 μM HNK).

Annotation	Fold enrichment	
	TRO	HNK
GO Biological Process		
Glycine betaine transport	11.33	-
Haem transport	11.33	11.99
Siderophore biosynthetic process	11.33	10.49
Siderophore-iron import into cell	10.20	5.99
Cellular response to iron ion	9.06	9.59
Zinc ion transport	8.50	-
Cysteine biosynthetic process	8.09	6.85
L-histidine biosynthetic process	7.55	-
Urea metabolic process	7.55	7.99
Cell redox homeostasis	7.21	8.72
Cobalt ion transport	6.29	-
Cellular oxidant detoxification	4.53	6.39
Response to oxidative stress	3.97	4.80
Nickel cation transport	3.33	-
Unclassified	0.67	0.75
Translation	0.11	-
Carbohydrate derivative biosynthetic process	0.10	-
Nucleoside phosphate biosynthetic process	<0.01	<0.01
Transmembrane transport	-	1.82
GO Molecular Function		
Cysteine synthase activity	11.33	11.99
Thioredoxin-disulphide reductase (NADPH) activity	11.33	11.99
Urease activator activity	11.33	11.99
Nickel cation binding	9.91	8.99
Modified amino acid transmembrane transporter activity	9.06	-
P-type ion transporter activity	9.06	9.59
Unclassified	0.53	0.65
RNA binding	<0.01	-
Thioredoxin peroxidase activity	-	11.99
GO Cellular Component		
Plasma membrane	1.56	1.49
Unclassified	0.78	0.79

TABLE S6 Significantly enriched GO terms (*p*-adj <0.05) of downregulated DEGs in *S. aureus* following exposure to high concentrations of chelant (1000 μM TRO or 100 μM HNK).

Annotation	Fold enrichment	
	TRO	HNK
GO Biological Process		
Nitrate assimilation	11.70	14.97
Anaerobic respiration	11.70	14.97
Lactose catabolic process via tagatose-6-phosphate	11.70	14.97
‘de novo' IMP biosynthetic process	10.72	13.72
Hexose transmembrane transport	8.77	11.23
L-arginine catabolic process	8.77	11.23
Carotenoid biosynthetic process	7.80	9.98
Monocarboxylic acid transport	6.68	8.55
Phosphate ion transport	6.50	13.31
Phosphoenolpyruvate-dependent sugar phosphotransferase system	5.67	8.16
Sodium ion transmembrane transport	4.92	-
Monosaccharide metabolic process	3.60	5.18
Unclassified	0.76	0.52
Protein metabolic process	0.30	-
Organic acid biosynthetic process	0.24	-
Cellular component organisation or biogenesis	0.23	0.19
Gene expression	0.11	<0.01
DNA metabolic process	0.10	-
RNA biosynthetic process	<0.01	-
Maltose transport	-	14.97
Maltodextrin transmembrane transport	-	14.97
Purine nucleobase biosynthetic process	-	8.98
Organic hydroxy compound transport	-	8.98
Carbohydrate derivative transport	-	6.24
Carboxylic acid transmembrane transport	-	4.49
Organophosphate catabolic process	-	3.74
Carbohydrate derivative catabolic process	-	3.58
Nucleic acid biosynthetic process	-	<0.01
GO Molecular Function		
Nitrate reductase activity	11.70	14.97
Oxidoreductase activity, acting on the CH-CH group of donors, quinone or related compound as acceptor	11.70	14.97
Phosphoribosylformylglycinamidine synthase activity	11.70	14.97
Protein-phosphocysteine-sugar phosphotransferase activity	8.35	11.76
Protein-N(PI)-phosphohistidine-sugar phosphotransferase activity	5.85	8.73
Sodium:proton antiporter activity	5.12	-
Kinase activity	2.39	2.75
Unclassified	0.70	0.54
Catalytic activity, acting on a nucleic acid	0.12	0.16
RNA binding	<0.01	<0.01
Disaccharide transmembrane transporter activity	-	14.97
Phosphate transmembrane transporter activity	-	12.83
GO Cellular Component		
Nitrate reductase complex	11.70	14.97

Plasma membrane	1.51	1.60
Unclassified	-	0.72

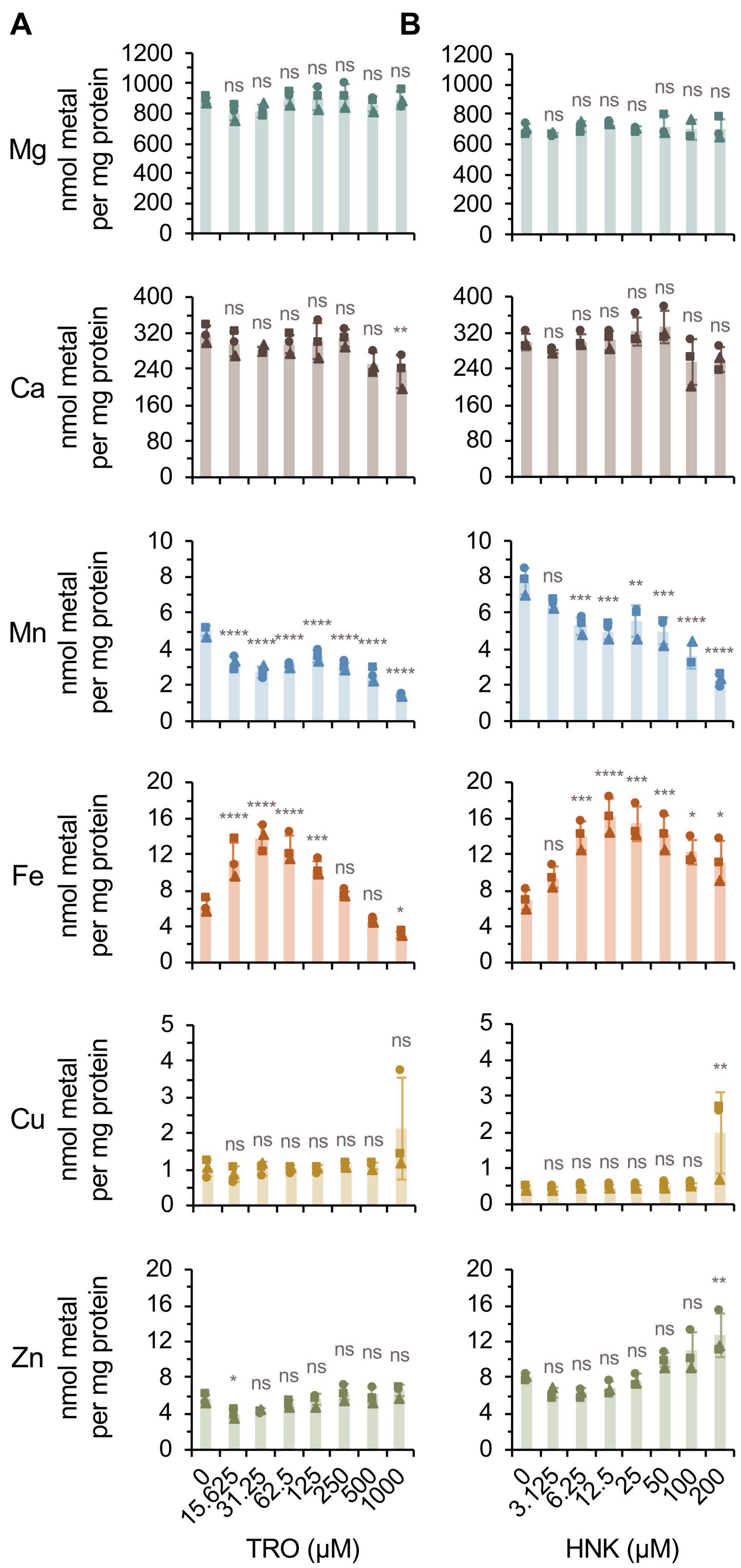

FIG S1 Effect of TRO and HNK on *B. subtilis* cellular metal levels. (A and B) Change in total cellular amounts of magnesium, calcium, manganese, iron, copper, and zinc in *B. subtilis* following treatment with TRO and HNK. Bacteria were cultured to early-mid log phase in 20 ml of LB before addition of chelant. ICP-MS was used to measure total cellular amounts of each metal and results are presented as nmol metal per mg total cellular protein. A one-way analysis of variance (ANOVA) with a post hoc Dunnett test was performed to compare each chelant concentration against the untreated control (n=3), *p <0.05, **p <0.01, ***p <0.001, ****p <0.0001, and ns = not significant.

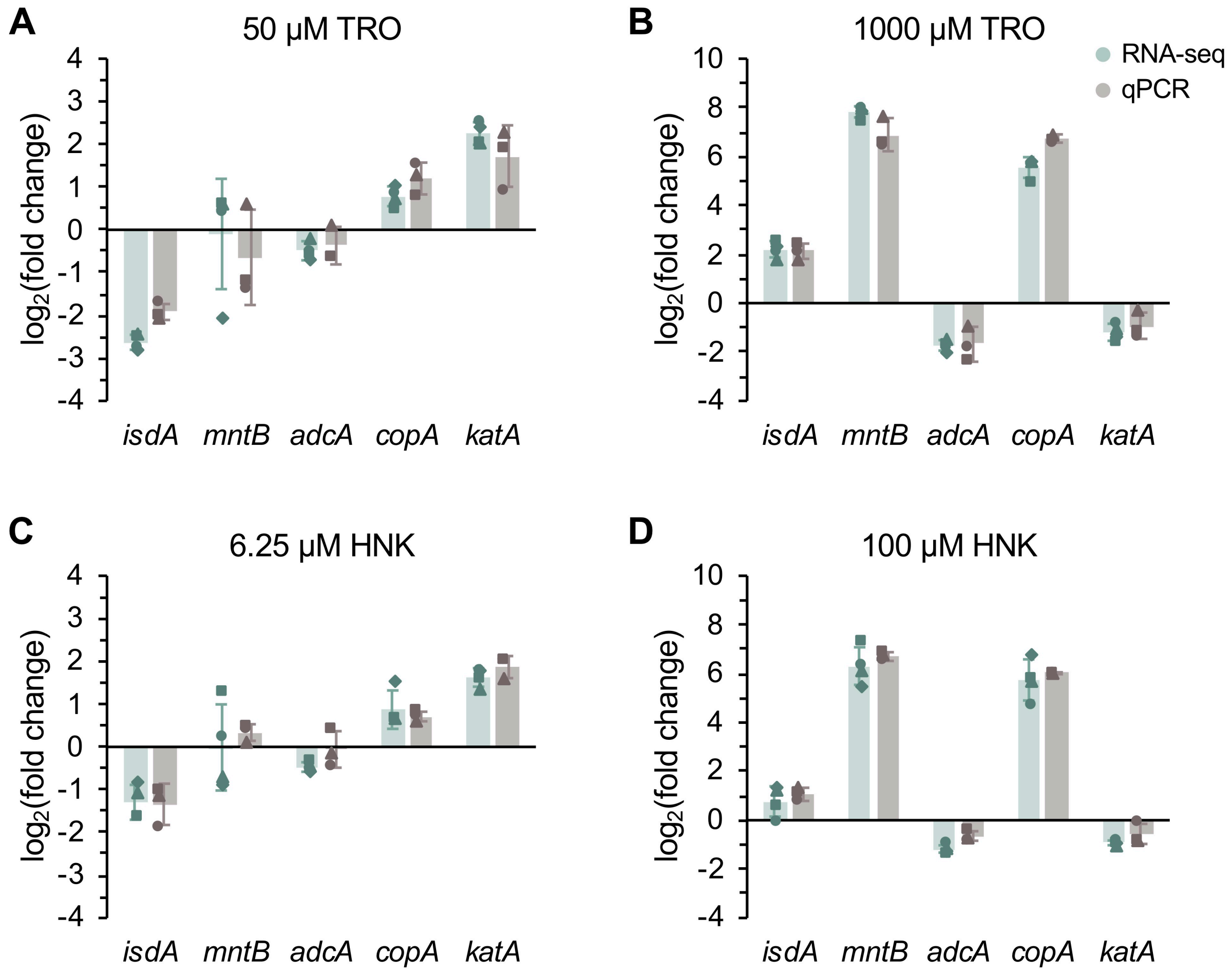

FIG S2 Validation of RNA-seq data for selected genes by RT-qPCR. Expression of DEGs identified in *S. aureus* FDA209P after exposure to 50 µM and 1000 µM TRO (A and B) or 6.25 µM and 100 µM HNK (C and D).

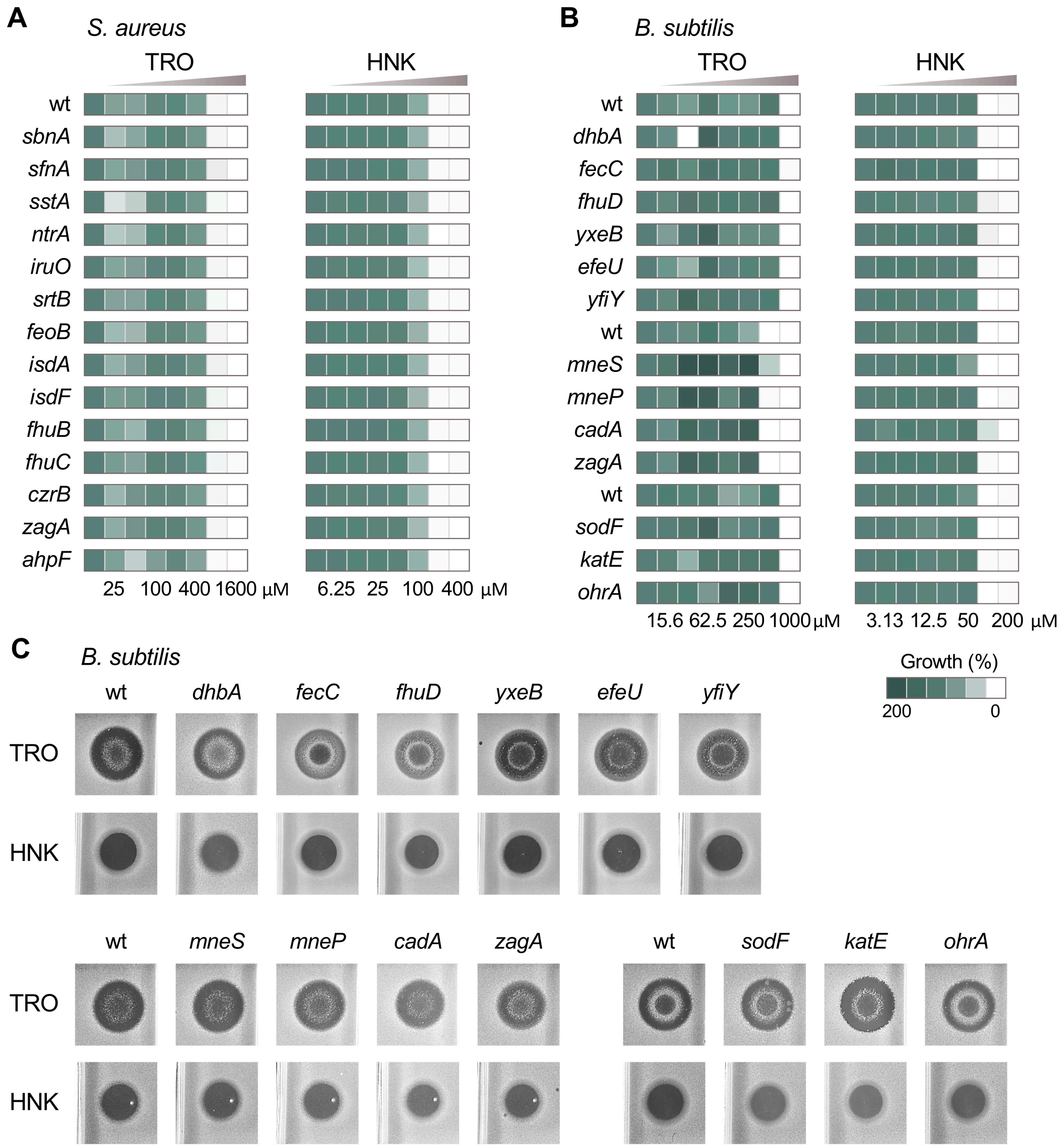

FIG S3 Antimicrobial activity of TRO and HNK against additional *S. aureus* and *B. subtilis* mutants deficient in selected metal transport systems and the oxidative stress response. (A and B) Mutant strains of *S. aureus* USA300 (*erm* insertions) and *B. subtilis* 168 (deletion-*kan* insertions) were cultured in 100 µl of LB, in the presence or absence of chelant, by shaking at 37°C for 16 h. OD_{600nm} was recorded, and percentage growth was determined relative to untreated controls. Data shows the mean percentage growth of three biological replicates (Supplemental Data File S1). (C) Samples of TRO and HNK (20 mM, 10 µl) were applied to soft agar overlays containing *B. subtilis*, which were incubated at 30°C for 20 h. Lighter areas show bacterial growth and darker areas show growth inhibition. Representative images are shown and two additional biological replicates produced similar results.

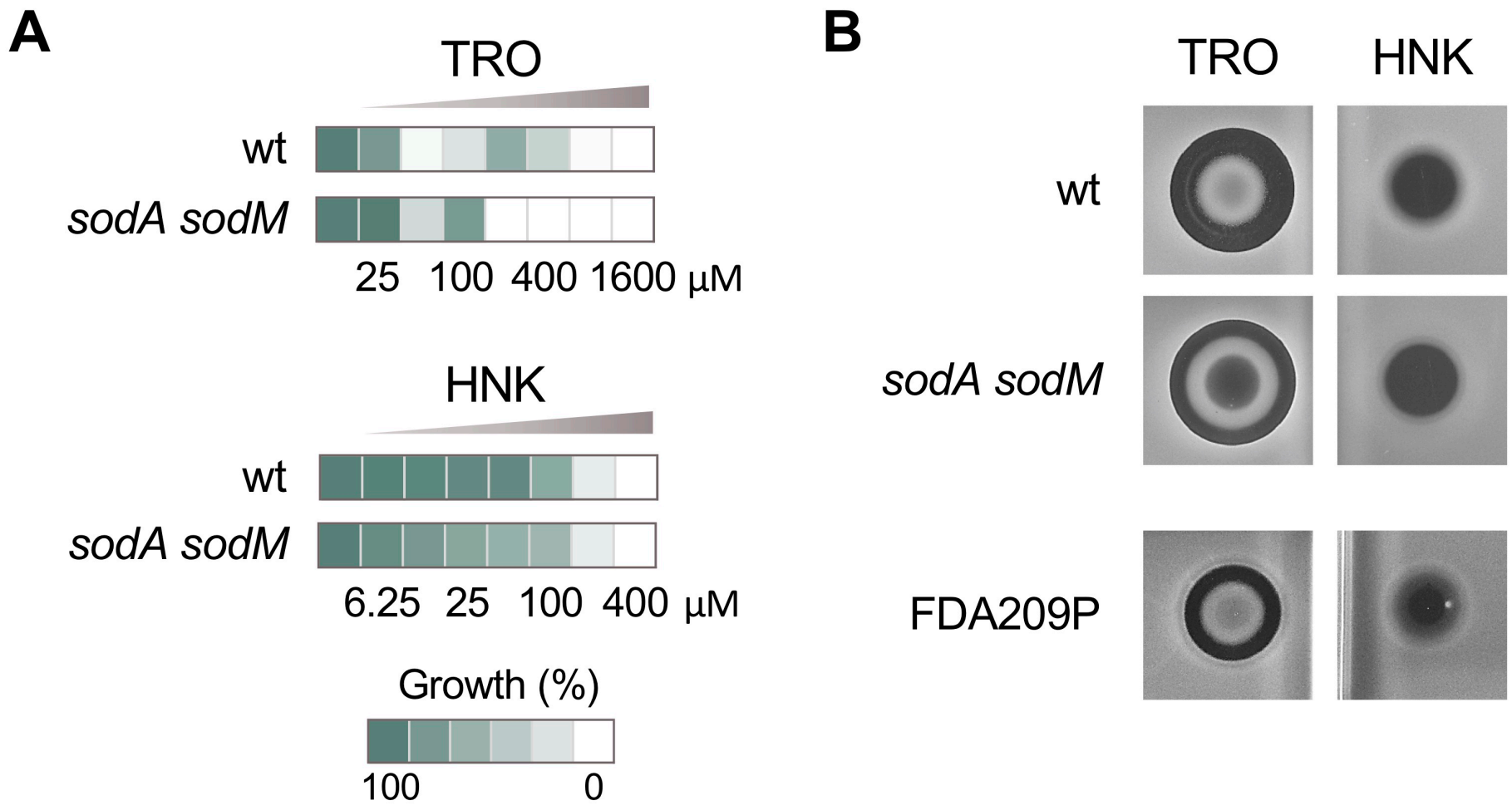

FIG S4 Susceptibility of *S. aureus* mutant lacking superoxide dismutases to TRO and HNK. (A) *S. aureus* SH1000 wt and *sodA*::*Tn917 sodM*::*tet* were cultured in 100 µl of LB, in the presence or absence of chelant, by shaking at 37°C for 16 h. OD_{600nm} was recorded, and percentage growth was determined relative to untreated controls. Data shows the mean percentage growth of three biological replicates (Supplemental Data File S1). (B) Samples of TRO and HNK (20 mM, 10 µl) were applied onto soft agar overlays containing *S. aureus*, which were incubated at 30°C for 20 h. Lighter areas show bacterial growth and darker areas show growth inhibition. Representative images are shown and two additional biological replicates produced similar results. An overlay with *S. aureus* FDA209P wt is included to facilitate comparisons with the SH1000 strains.

References

1. Singh M, Sasaki T, Matsuo M, Morimoto Y, Aiba Y, Hiramatsu K. 2015. Complete genome sequence of the drug-naive classical *Staphylococcus aureus* strain FDA209P. Genome Announc. 3:e01343-15.
2. Horsburgh MJ, Aish JL, White IJ, Shaw L, Lithgow JK, Foster SJ. 2002. σ^B modulates virulence determinant expression and stress resistance: characterization of a functional *rsbU* strain derived from *Staphylococcus aureus* 8325-4. J. Bacteriol. 184:5457-5467.
3. Karavolos, M. H., Horsburgh, M., Ingham, E., Foster, S. J. 2003. Role and regulation of the superoxide dismutases of *Staphylococcus aureus*. Microbiology (Reading). 149:2749-2758.
4. Barbe V, Cruveiller S, Kunst F, Lenoble P, Meurice G, Sekowska A, Vallenet D, Wang T, Moszer I, Médigue C, Danchin A. 2009. From a consortium sequence to a unified sequence: the *Bacillus subtilis* 168 reference genome a decade later. Microbiology (Reading). 155:1758-1775.
